# A Prosthetic Socket with Active Volume Compensation for Amputated Lower Limb

**DOI:** 10.3390/s21020407

**Published:** 2021-01-08

**Authors:** Ji-Hyeon Seo, Hyuk-Jin Lee, Dong-Wook Seo, Dong-Kyu Lee, Oh-Won Kwon, Moon-Kyu Kwak, Kang-Ho Lee

**Affiliations:** 1 Daegu Research Center for Medical Devices, Korea Institute of Machinery and Materials, Daegu 42994, Korea; gch02236@kimm.re.kr (J.-H.S.); machlhj@kimm.re.kr (H.-J.L.); dongkyu@kimm.re.kr (D.-K.L.); owkwon@kimm.re.kr (O.-W.K.); 2 School of Mechanical Engineering, College of Engineering, Kyungpook National University, Daegu 41566, Korea; mkkwak@knu.ac.kr; 3 Department of Radio Communication Engineering/Interdisciplinary Major of Maritime AI Convergence, Korea Maritime and Ocean University, Busan 49112, Korea; dwseo@kmou.ac.kr

**Keywords:** lower limb prosthesis, prosthetic socket, active control, volume compensation, air bladder, air flow, 3-way pneumatic valve

## Abstract

Typically, the actual volume of the residual limb changes over time. This causes the prosthesis to not fit, and then pain and skin disease. In this study, a prosthetic socket was developed to compensate for the volume change of the residual limb. Using an inflatable air bladder, the proposed socket monitors the pressure in the socket and keeps the pressure distribution uniform and constant while walking. The socket has three air bladders on anterior and posterior tibia areas, a latching type 3-way pneumatic valve and a portable control device. In the paper, the mechanical properties of the air bladder were investigated, and the electromagnetic analysis was performed to design the pneumatic valve. The controller is based on a hysteresis control algorithm with a closed loop, which keeps the pressure in the socket close to the initial set point over a long period of time. In experiments, the proposed prosthesis was tested through the gait simulator that can imitate a human’s gait cycle. The active volume compensation of the socket was successfully verified during repetitive gait cycle using the weight loads of 50, 70, and 90 kg and the residual limb model with a variety of volumes. It was confirmed that the pressure of the residual limb recovered to the initial state through the active control. The pressure inside the socket had a steady state error of less than 0.75% even if the volume of the residual limb was changed from −7% to +7%.

## 1. Introduction

The amputated patient removed parts of the body for a variety of reasons, including trauma, tumor, peripheral arterial disease, infection, and diabetic foot ulcer [1,2,3]. Unfortunately, the number of patients with amputated limb is increasing every year [2,4]. As the number of amputated patients increases, so does the need for patient-specific prosthesis. Prosthesis plays an important role in helping the patient perform a variety of daily activities naturally [3,5]. In particular, successful rehabilitation for patients with amputated lower limb is associated with the comfort and fit of the prosthesis while walking. Prosthesis fitting is mainly determined by socket design and suspension system. The socket design was classified into Specific Surface Bearing (SSB) sockets in the early 1950s and Total Surface Bearing (TSB) sockets in the 1980s according to the size of the loading area at the residual limb. TSB sockets have been widely used as they avoid high local stresses and improve comfort and fit [6]. As a socket suspension system, the liner is typically used in conjunction with a distal locking mechanism or an air evacuation system. The liner increases normally the stability between the residual limb and the socket. In particular, the suspension system based on the vacuum-assisted socket allows a better fitting in comparison with distal locking mechanism [6].

Recent prosthesis research studies have focused on volume fluctuations as a key parameter affecting the socket’s efficiency and acceptability. Typically, the volume of the residual lower limb can be changed from −11% to 7% per day [6,7]. The volume change is different depending on the patient’s daily activity level, eating habits, and so on [8,9]. Due to the volume change, contact with the prosthetic socket is not optimized and the internal pressure is concentrated in the localized area of the residual limb. It causes problems such as the bell clapping effect of a lateral displacement and the piston effect of vertical displacement, resulting in pain and skin disease [10,11]. In fact, skin disease occurs in 63–82% of patients with amputated lower limb, which has been reported to increase the rate of prosthesis disposal [6]. For most amputation patients, it is difficult to replace sockets frequently due to the burden of time and money, so supplements such as socks or pads have been used to compensate for the volume changes of the residual limb. However, these supplements are difficult to have a uniform pressure distribution and rather they sometimes cause excessive pressure on the residual limb. Recently, studies on the internal pressure generated between the residual limb and the socket have been conducted [12,13,14,15,16]. Some sockets change the volume in the prosthetic socket by manually tightening or releasing it using a dial button, clamps or lacing system [6,9]. Using these sockets, the patients can reduce excessive pressure applied to the residual limb compared to inserting socks or pads. However, this always requires a user’s control, not automatically, and may bring patients to exceed in tightening their socket due to the desire of maximizing the fitting and stability of their prosthesis [6]. In addition, it is difficult to compensate for volume changes in specific parts of the residual limb. Other sockets automatically adjust socket fit using an inflatable air bladder [16,17] or motor-based movement [18]. The socket in [16] has included pneumatic actuator inserts for interface pressure mapping and fit improvement. However, its control system for actuation is bulky to be used as a wearable device and is still at the laboratory level. The air pneumatic suspension system [17] is portable and contributes to reducing the pressure concentration around limb, but it is not sufficiently validated to compensate for various volume changes of the residual limb. Sockets with motor-driven panels [18] maintain socket fit while measuring the distance between the residual limb and socket. However, the rigid panel is difficult to adhere to the curved limb, and it should be portable by improving the power consumption and weight due to the motor.

In this paper, we propose a prosthetic socket system that actively compensates for the volume change of the residual limb. The proposed socket uses an inflatable air bladder to monitor the pressure in the socket and keep the pressure distribution uniform and constant while walking. Unlike conventional studies, our system focuses only on long-term dynamic situations where the volume of the lower limb changes over a long period of time. This allows the control device including an air pump, a valve, a circuit board and a Li-Po battery to be minimized enough to attach to the socket case. Our portable device provides a good socket fit in spite of volume fluctuations in limbs. In this study, the prosthetic socket was tested only using the gait simulator to verify its feasibility, not a clinical test. We developed the gait simulator that can imitate a stance phase in the gait cycle, and a closed-loop algorithm for active volume compensation. In experiments, we investigated the performance of the prototype and demonstrated active compensation of the residual limb volume. The proposed socket system is expected to help patients with amputated limbs feel comfortable and improve their quality of life.

## 2. Materials and Methods

### 2.1. System Structure and Operation Principle

**Figure 1:**
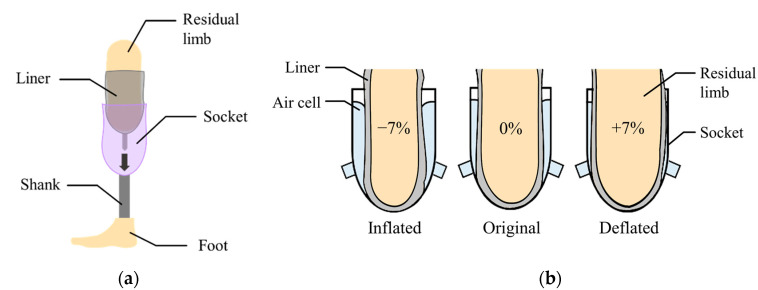
(**a**) The components of a lower limb prosthesis; (**b**) the volume compensation by inflation or deflation of the air bladder.

Figure 1a shows a transtibial prosthesis for patient with a lower limb amputation. The socket supports the body weight while distributing the forces through the residual limb. The liner, typically made of silicon, primarily protects the residual limb that is sensitive to irritation.

In this paper, the air bladder is located between the liner and the prosthetic socket. The air bladder detects the pressure caused by the compression between the residual limb and the socket in real time, and then changes its volume to compensate for the change in volume of residual limb. Figure 1b shows the concept of the active volume-compensating socket. As the volume of the residual limb increases, the pressure in the air bladder increases and then the air bladder deflates to reduce the pressure. Additionally, as the residual limb volume decreases, the air bladder inflates to increase the pressure. In the proposed socket system, the pressure in the socket is expected to return to its original state despite the change in volume of residual limb. While wearing the prosthetic socket, the pressure between the residual limb and the socket remains the same.

**Figure 2:**
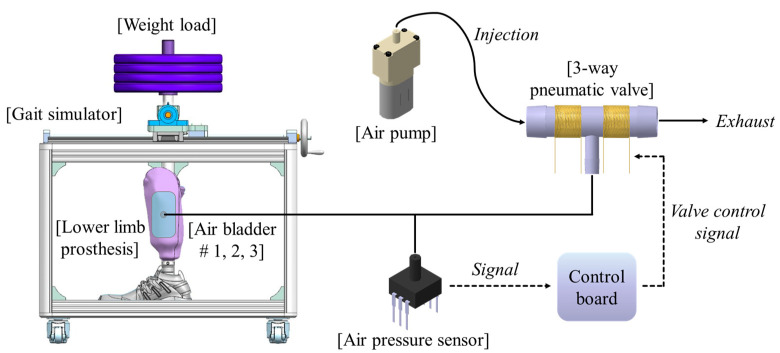
The conceptual diagram of the proposed socket system to actively compensate for the volume change in residual limb.

**Figure 3:**
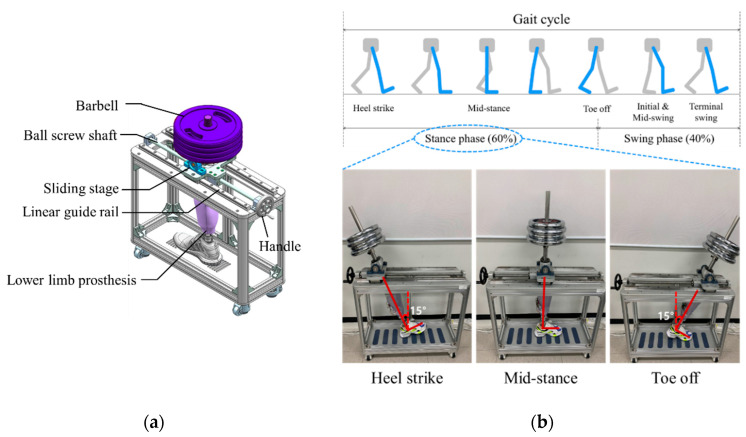
(**a**) The design of the gait simulator, and (**b**) the gait simulation for the stance phase of the gait cycle.

Figure 2 shows the proposed socket system to actively compensate for the volume change of the residual limb. The system consists of a gait simulator, a lower limb prosthesis, air bladders located in the socket, an air pump, a 3-way pneumatic valve, an air pressure sensor, and a control board. As the proof of concept, the proposed socket was tested only using the gait simulator, not a clinical test. The gait simulator can simulate a human’s gait with weight loads. The 3-way pneumatic valve supervises the airflow into and out of the air bladder. When the 3-way pneumatic valve opens to the injection side, air is injected from the air pump to fill the air bladder. Conversely, when the 3-way pneumatic valve is opened to the exhaust side, the air filled in the air bladder is released into atmosphere. The control board measures the pressure in the air bladder using the air pressure sensor and controls ON/OFF signals for the 3-way pneumatic valve. The gait simulator is detailed in Figure 3a. The gait simulator is based on a linear motion system. The linear motion system consists of a ball screw shaft, a sliding stage with nut and a linear guide rail. The sliding stage to which the prosthesis was connected was designed to move along a linear guide rail. By manually rotating the handle, the ball screw moves the prosthesis back and forth. The swing angle of the implemented prosthesis is ±15 degrees. In addition, the sliding stage supports the stacked barbells. Here, various weights such as 50, 70, 90 kg were used to indirectly express the body weight. The gait cycle is typically divided into a stance phase and a swing phase [19,20,21]. The stance phase is a state in which weight is loaded while the foot touches the ground, and in the swing phase, the lower limb moves forward away from the ground, and the weight is loaded on the opposite lower limb. The gait simulator only expresses the stance phase of the gait cycle, not the swing phase. This is because the pressure in prosthetic socket is not affected during the swing phase. The gait simulator successfully represents three stages in the stance phase: heel strike, mid-stance and toe off, as shown in Figure 3.

### 2.2. Characterization of an Inflatable Air Bladder

**Figure 4:**
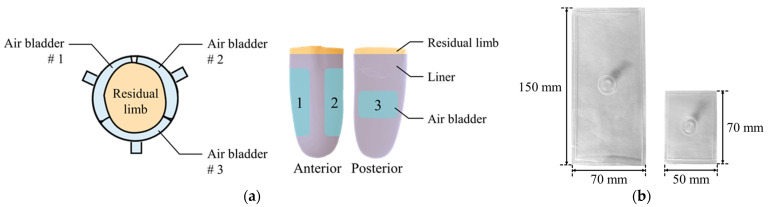
(**a**) The locations of the air bladders in the prosthetic socket; (**b**) the appearance of the air bladders.

Air bladder is located in the prosthetic socket to compensate for the volume change of the residual limb. The residual limb should be centered on the prosthetic socket even if the volume of the residual limb changes. Therefore, as shown in Figure 4a, the air bladders were located to surround the residual limb. This arrangement reduces the movement of the residual limb in the prosthetic socket. The three air bladders were located on anterior and posterior tibia areas, which is the minimum number that allows the residual limb to be centered. Air bladder #1 and #2 at the anterior tibia have a size of 150 (L) × 70 (W) mm, and air bladder #3 at posterior tibia has a size of 70 (L) × 50 (W) mm. Using TPU (Thermoplastic Polyurethane) material with a thickness of 0.5 mm, the air bladder was made to fit the residual limb as shown in Figure 4b.

**Figure 5:**
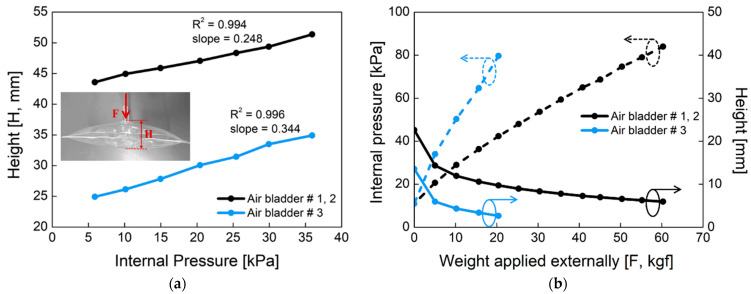
The mechanical properties of the air bladder. (**a**) Changes in the height of the air bladder according to the internal pressure; (**b**) changes in the internal pressure (dotted line) and height (full line) of the air bladder as the applied weight varies, when initial pressure in the air bladder is 11 kPa.

Figure 5 shows the mechanical properties of the proposed air bladder. In Figure 5a, changes in the height of the air bladder were investigated according to the different internal pressure. For air bladder #1(or #2) and #3, the heights of the air bladders were changed linearly as the internal pressure increased, having slopes of 0.248 and 0.344, respectively. The maximum height was about 50 mm and 35 mm for air bladder #1(or #2) and #3, respectively. In Figure 5b, changes in the internal pressure (dotted lines) and height (full lines) of the air bladder were investigated according to the weight applied externally. As the pressing weight increased, the internal pressure started to increase from the initial pressure of 11 kPa. The small air bladder #3 had a larger slope in internal pressure than that of air bladder #1(or #2). As the pressing weight increased, the heights of the air bladders decreased and kept their heights at the saturated point. The air bladder #1(or #2) showed the internal pressure of 80 kPa at 60 kgf weight load, and the air bladder #3 showed 80 kPa at 20 kgf. Here, the maximum allowable weight was determined as a percentage of the air bladder in the entire socket, assuming that a person with a weight of 90 kg wore the prosthesis.

### 2.3. Design of the 3-Way Pneumatic Valve

**Figure 6:**
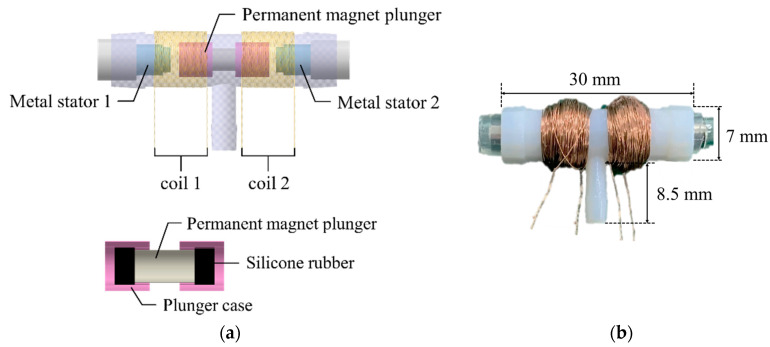
(**a**) The cross section of 3-way pneumatic valve; (**b**) The appearance of the pneumatic valve.

**Figure 7:**
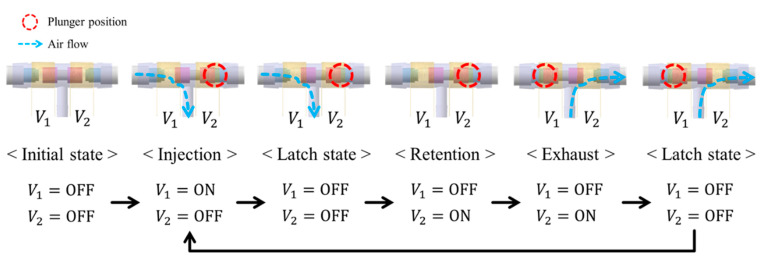
Operating process of the 3-way pneumatic valve.

The 3-way pneumatic valve was designed to inject or exhaust air through the air bladder as shown in Figure 6a. It includes a bi-directional latching solenoid that consists of two coils, metal stators at both ends and a permanent magnet plunger. Typically, latching solenoid uses a magnetic attraction to maintain the plunger’s position without the electric power supply [22,23]. Therefore, the latching solenoid is suitable for portable applications with the battery power. Two coils placed in series alternatively generate two magnetic fields, which induces the plunger to move between two metal stators. The plunger begins to move by an energized coil, and then it keeps its position in the one-sided location by the magnetic attraction force. This solenoid valve only needs a transient current to move the plunger towards the metal stator, and no conducting current after the plunger moves. Silicon rubber is attached to both ends of the permanent magnet to keep the gap between the plunger and the metal stator. Figure 6b shows the appearance of the 3-way pneumatic valve. One side of the 3-way pneumatic valve is connected to the air pump to supply air into the air bladder, and the other to exhaust air into atmosphere. Figure 7 shows the operating process of the pneumatic valve. Initially, no voltage is supplied to the two solenoid coils.

When a voltage *V*_1_ is supplied to the left coil, the permanent magnet plunger moves to the right position by repulsive force and then air is injected into the air bladder. After the plunger moves, it keeps its position to the metal stator by magnetic attraction force without supplying power. When voltage *V*_2_ is supplied to the right coil, the plunger moves to the left position and then air is exhausted into atmosphere. After that, it stays in a latching state.

**Figure 8:**
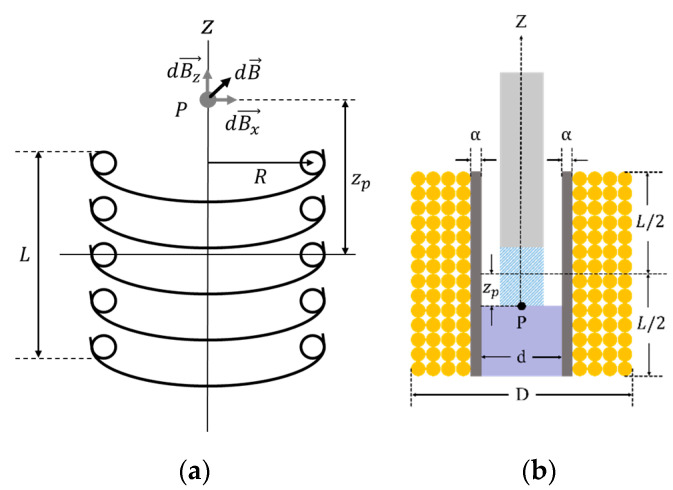
(**a**) The electromagnetic field diagram caused by circular coils (P is the point of interest for electromagnetic analysis; $\vec{B_{z}}$, $\vec{B_{x}}$ and $\vec{B}$ are the electromagnetic field in the z, x-direction and sum-direction of z and x as the solenoid axis, respectively; *L* is the length of solenoid; *R* is the radius of circular coil; $z_{p}$ is the distance from the center of the solenoid to point); (**b**) the cross-sectional view for solenoid design (d and D are the inner diameter and the outer diameter of solenoid, respectively; α is the thickness of the solenoid wall).

To design a latching solenoid with a permanent magnet plunger, one requirement must be established. The strength of the electromagnetic field generated by the solenoid must be strong enough to remove the latched plunger attracted to the metal. That is, the electromagnetic force of the solenoid must be greater than the pull force of the permanent magnet. So, the electromagnetic field was analyzed at the point where the permanent magnet plunger is held on the metal. The electromagnetic force of the solenoid is determined by a current through the coil, a length and cross-sectional area of the solenoid, and number of coil turns [24]. In Figure 8a, the electromagnetic field produced by solenoid coil at point *P* is as follows [25]:

(1) $$\vec{B_{z}}=\frac{\mu_{0}iR^{2}}{4\pi {\left(z_{p}{}^{2}+R^{2}\right)}^{3/2}}\int_{0}^{2\pi }d\theta \hat{k}=\frac{\mu_{0}iR^{2}}{2{\left(z_{p}{}^{2}+R^{2}\right)}^{3/2}}\hat{k}$$

where $\vec{B_{z}}$ is the electromagnetic field in the z-direction coordinate as the solenoid axis, and $\hat{k}$ is its unit vector. $\mu_{0}$ and i are the permeability of vacuum and the coil current, respectively. $z_{p}$ is the distance from the center of the solenoid to point P.

Using Equation (1) and the design parameters of the solenoid in Figure 8b, the electromagnetic field at point P becomes [24]

(2) $$B_{z}=\frac{\mu_{0}iN}{2L\left(D-d\right)}\left[\left(L+2z_{p}\right)\ln\left\{\frac{D+\sqrt{D^{2}+{\left(L+2z_{p}\right)}^{2}}}{d+\sqrt{d^{2}+{\left(L+2z_{p}\right)}^{2}}}\right\}+\left(L-2z_{p}\right)\ln\left\{\frac{D+\sqrt{D^{2}+{\left(L-2z_{p}\right)}^{2}}}{d+\sqrt{d^{2}+{\left(L-2z_{p}\right)}^{2}}}\right\}\right]$$

where d, D, N and L are the inner diameter, the outer diameter, the number of coil turns and the length of solenoid, respectively.

Here, the coil current i varies with the number of coil turns and is defined as follows:

(3) $$i=\frac{V}{R_{eq}\left(i\right)+R_{coil}\left(N\right)}$$

where V is the supply voltage and $R_{eq}$ is the equivalent resistance caused by the coil current. The $R_{eq}$ includes ON resistance of the MOSFET (Metal Oxide Semiconductor Field Effect Transistor) switch, parasitic components on the PCB (Printed Circuit Board) and so on. $R_{coil}$ is the coil resistance.

Since the latching solenoid uses a permanent magnet plunger, the electromagnetic force generated is expressed as [24]

(4) $$F_{z}=\int \sigma_{m}B_{sol,z}\;ds=\frac{B_{r}}{\mu_{0}}B_{sol,z}\int_{0}^{2\pi }\int_{0}^{\frac{d}{2}+\alpha }r\;dr\;d\phi =\frac{B_{r}B_{sol,z}}{\mu_{0}}\pi {\left(\frac{d}{2}+\alpha \right)}^{2}$$

where the electromagnetic field by the solenoid, $B_{sol,z}$, is equal to Equation (2), and $\sigma_{m}$ is equivalent surface charge density. $B_{r}$ is the remanence or residual flux density of the permanent magnet. α is the thickness of the solenoid wall.

The electromagnetic force is proportional to the magnetic field of solenoid and magnet. Here, $B_{sol,z}$ changes according to the variables of coil turns N and coil current i, and other parameters can be considered as constants determined by the solenoid structure.

**Figure 9:**
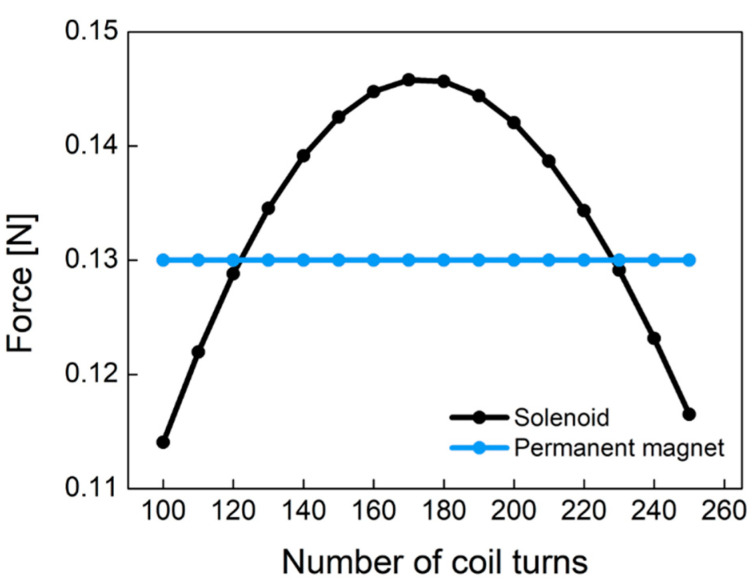
The comparison of the permanent magnet force and the solenoid force according to the different number of coil turns.

Figure 9 shows the electromagnetic force of Equation (4) caused by the solenoid and the pull force by the permanent magnet according to the different number of coil turns. Permanent magnet (NdFeB) has a constant pull force of 0.13 N regardless of the number of coil turns. This figure was obtained from experiments with the plunger used in this study. As the number of coil turns increases, the electromagnetic force increases at low turns of coil. However, as the number of coil turns increases, the electromagnetic force begins to decrease because the coil resistance and non-linear conduction current are dominant at high turns of coil. The electromagnetic force of the designed solenoid has a convex curved point. In the latching solenoid, the electromagnetic force ($F_{solenoid}$) by the solenoid has to be larger than the pull force ($F_{permanent\;magnet}$) of the permanent magnet in order to remove the permanent magnet plunger attracted to the metal. In Figure 9, it is found that the coil turns of 170 is the optimum point. As a result, we designed the pneumatic valve in the socket system with 170 coil turns.

### 2.4. Closed-Loop Control Strategy

**Figure 10:**
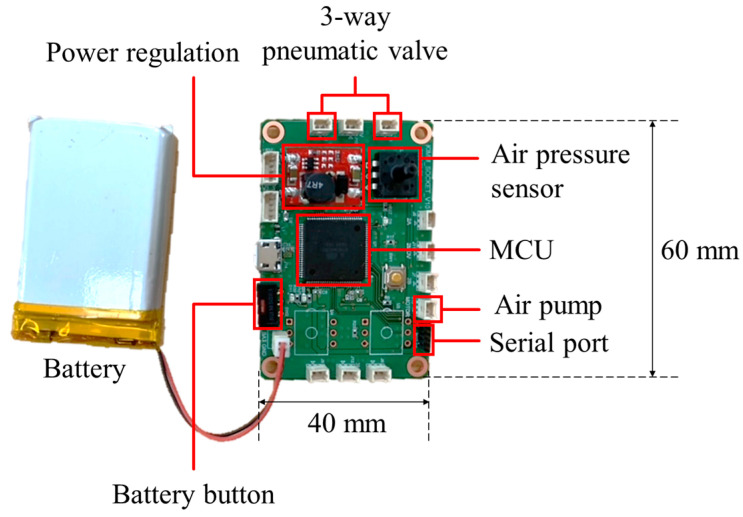
The control board for socket system with volume compensation function.

The control board was manufactured to measure the internal pressure of the air bladder and determine the direction of the pneumatic valve. Figure 10 shows the control board with a size of 60 (L) × 40 (W) mm, which used a microcontroller of ATmega2560 (Microchip Technology Inc., Chandler, AZ, USA) and powered a 3.7 V Li-Po battery.

**Figure 11:**
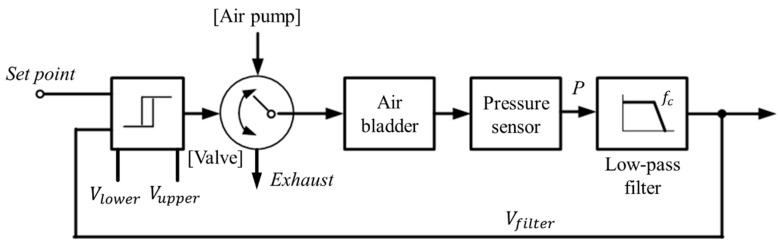
The hysteresis control algorithm with a closed loop.

**Figure 12:**
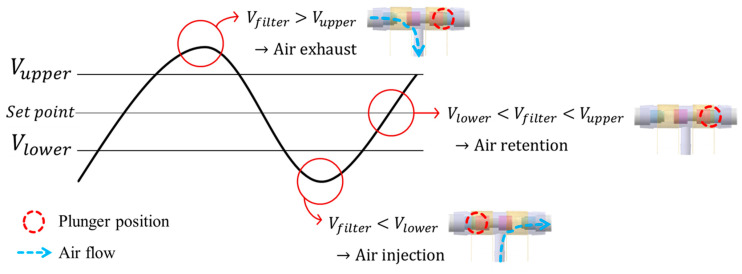
Description of operation by the hysteresis control algorithm.

While wearing the prosthetic socket, the volume of the lower limbs changes over a long period of time. For this reason, the controller in Figure 11 operates very slowly to complete the volume compensation work. The proposed algorithm is based on the hysteresis control method, which keeps the pressure signal close to the initial set point by observing for a long time. Hysteresis control, unlike the PID (Proportional–Integral–Derivative) control method, is suitable for slower, simpler, and less precise operation [26,27,28]. The pressure of the air bladder is measured using the air pressure sensor, and the final signal *V_filter_* is generated through the low-pass filter. Low-pass filter eliminates the noise signal and makes the system a slow operation. Here, cutoff frequency *f_c_* of the low-pass filter depends on walking speed. The *V_filter_* is fed back and compared to the limit values. The limit values of hysteresis control are defined as *V_upper_* and *V_lower_*, which have a fixed offset from the set point. Here, if the difference between *V_upper_* and *V_lower_* is very large, the volume compensation error is large. On the other hand, if the difference is very small, air injection or exhaust occurs frequently, resulting in increased power consumption and excessive noise in the system. In our study, the fixed offset of ±3.5 kPa between the upper and lower limit was found optimally. In Figure 12, when the *V_filter_* is larger than *V_upper_*, air is exhausted into atmosphere, and when the *V_filter_* is smaller than *V_lower_*, air is injected into air bladder. The air in the bladder remains without any addition and reduction when the *V_filter_* is between the *V_upper_* and *V_lower_*.

## 3. Results and Discussion

### 3.1. Leg Limb Model with Volume Changes

**Figure 13:**
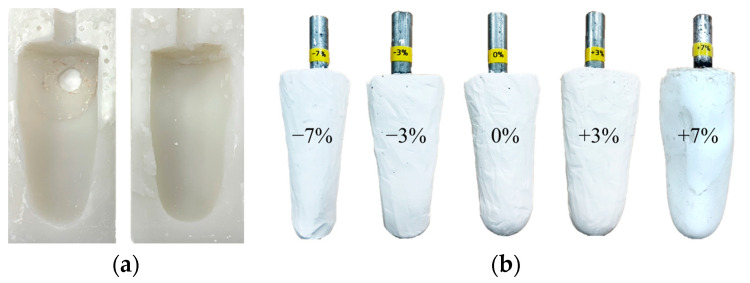
(**a**) The silicone mold for residual limb model; (**b**) the residual limb model with various volume changes of −7%, −3%, 0%, +3% and +7%.

The volume of the residual limb may vary in a single day due to activity levels. Since the socket proposed in this paper is evaluated using the gait simulator, a lower limb model with various volumes is required. First, a silicon mold was made by modeling the patient’s right leg amputation, as shown in Figure 13a. Then, using a silicon mold, various plaster models of the residual limb were produced with volume changes of −7%, −3%, 0%, +3% and +7%, as shown in Figure 13b. The range of the change in limb volume was determined by taking into account the actual volume change of the patient wearing the prosthetic leg [6].

### 3.2. Performance of a Closed-Loop Control

In Figure 11, the proposed system manages the internal pressure of the prosthetic socket using hysteresis control with a closed loop. The control performance was evaluated by the response time when the air bladder with no load was connected to the controller. The rise time of output signal *V_filter_* was measured when the set point pressure was changed from 6.5 kPa to 16.5 kPa in a step of 10 kPa, where 6.5 kPa is the minimum pressure at which pressure changes can be observed. The rise time was defined as the time it takes to reach from 10% to 90% of the step pressure.

**Figure 14:**
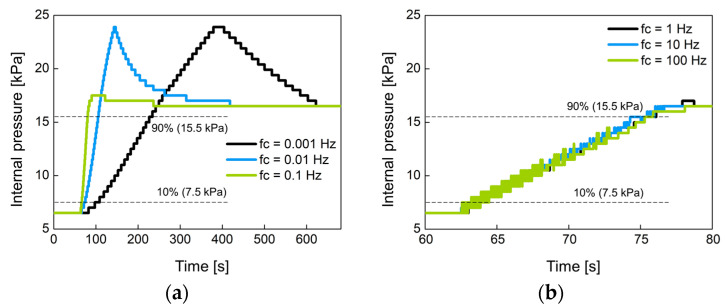

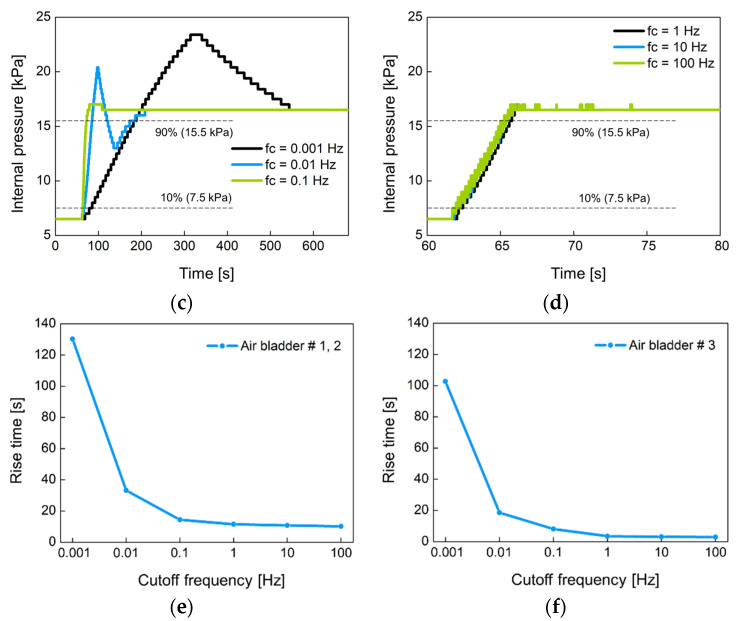
The change of the internal pressure when the set point pressure was changed from 6.5 kPa to 16.5 kPa in 10 kPa step at (**a**) low and (**b**) high cutoff frequencies for large air bladder #1 (or #2); (**c**) low and (**d**) high cutoff frequencies for small air bladder #3; the rise time in terms of cutoff frequency for (**e**) large air bladder #1(or #2) and (**f**) small air bladder #3.

Figure 14 shows the change of the internal pressure with the cutoff frequency *f_c_* of the low-pass filter used. The *f_c_* was set to 0.001 Hz, 0.01 Hz, 0.1 Hz, 1 Hz, 10 Hz, and 100 Hz. Figure 14a,b were experimented with large-sized air bladder #1(or #2) and small-sized air bladder #3, respectively. Low *f_c_* indicates slow control, so the rise time at low *f_c_* is greater than at high *f_c_*. Figure 14c,d show the rise time for each air bladder in terms of cutoff frequency. Small air bladder has a faster rise time than large air bladder. It was observed that the rising time hardly changed at cutoff frequencies over 1 Hz. The rise time was 10.2 s for large air bladder and 2.9 s for small air bladder. These rise times are sufficient to control the internal pressure of the socket because the volume of the lower limb changes over a long period of time.

**Figure 15:**
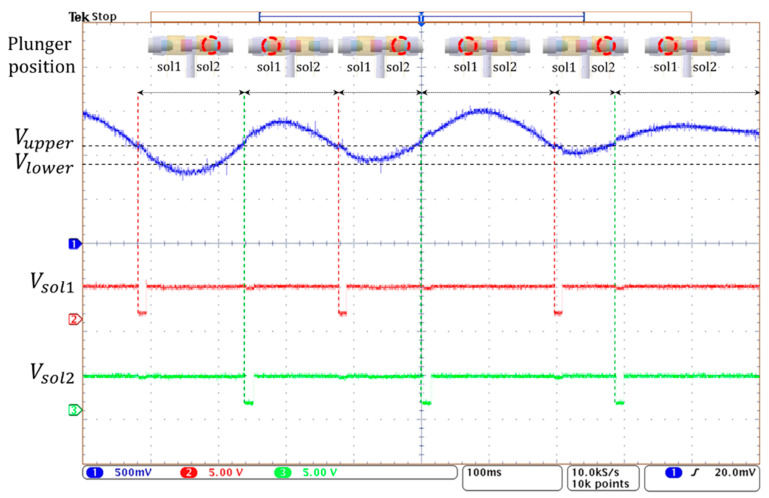
The operating waveform of 3-way pneumatic valve with hysteresis control.

Figure 15 shows the operation of the 3-way pneumatic valve with hysteresis control. After the random pressure signal (blue line) is compared to the limit values of *V_upper_* and *V_lower_*, the valve control signals of *V_sol1_* and *V_sol2_* (red and green line) of active low are generated to move the solenoid plunger. When the pressure signal is greater than *V_upper_*, the right coil of the solenoid is activated, and the plunger moves to the left. During this period, the air in the bladder is released into the atmosphere and the air pump does not work. When the pressure signal is below *V_upper_*, the left solenoid coil is activated and then the plunger moves to the right. Here, when the pressure signal is between the *V_upper_* and *V_lower_*, the air remains unchanged. When the pressure signal is below *V_lower_*, the air pump starts to work, and the air is injected into the air bladder. In Figure 15, the time it takes to move the plunger is about 10 ms, which is much shorter compared to the time the solenoid is in a latching state. Therefore, the latching solenoid used in the study can reduce power consumption and heat generation.

### 3.3. Verification of Closed-Loop Control during Gait Cycle

**Figure 16:**
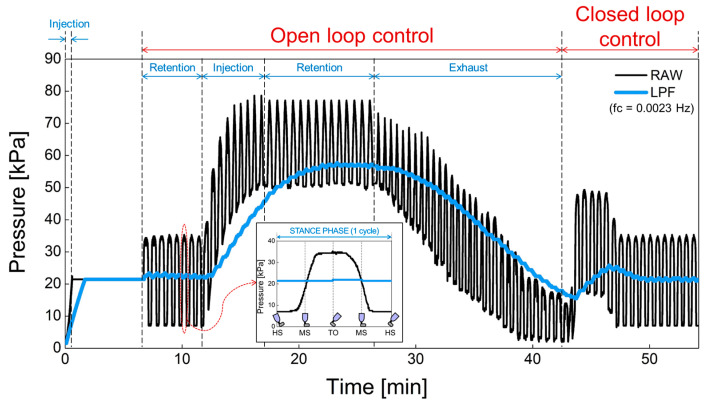
The open and closed loop control in a prosthesis with an air bladder inserted.

The proposed prosthesis performs repetitive walking motions through the gait simulator. In Figure 16, (1) the operation of the air bladder, (2) the pressure changes due to air injection or exhaust and, (3) the feedback operation of the pressure was verified using a prosthesis with an air bladder inserted. The black line is the raw data detected by the pressure sensor before low-pass filtering. In addition, the blue line is the pressure signal obtained through the low-pass filter. Here, we used the cutoff frequency for low-pass filter of 0.0023 Hz because the swing of the prosthesis in the gait simulator is very slow due to manual manipulation. In the clinical test, the gait speed varies according to the patient’s walking pattern or habit, but the filter needs to be designed with a cutoff frequency of less than 0.1 ~ 0.01 Hz, taking into account the normal gait speed of a person. The low cutoff frequency is suitable because our system responds to the lower limb volume that changes over a long period of time. This cutoff frequency does not need to be controlled automatically, and we think it will be appropriate to find a fixed value through clinical test in the future. In open-loop control, the sequence of operations is the retention, injection, retention, and exhaust of air. The pressure increased when the air was injected, and the pressure decreased when the air was exhausted. In addition, the pressure was retained in absence of air injection and exhaust. In closed-loop control, the pressure signal is kept close to the initial set point. The proposed prosthetic socket is observed to function normally in open and closed loop control. Actually, the pressure set point is a subjective parameter that can be adjusted according to the patient’s level of comfort, as the pain caused by the residual limb features such as bony prominences is different for each patient. In future work, the patients can manually set the initial setting value when they feel most comfortable while wearing the socket. This pressure set point can be changed by the user from time to time. 

### 3.4. Active Volume Compensation during Gait Cycle

**Figure 17:**
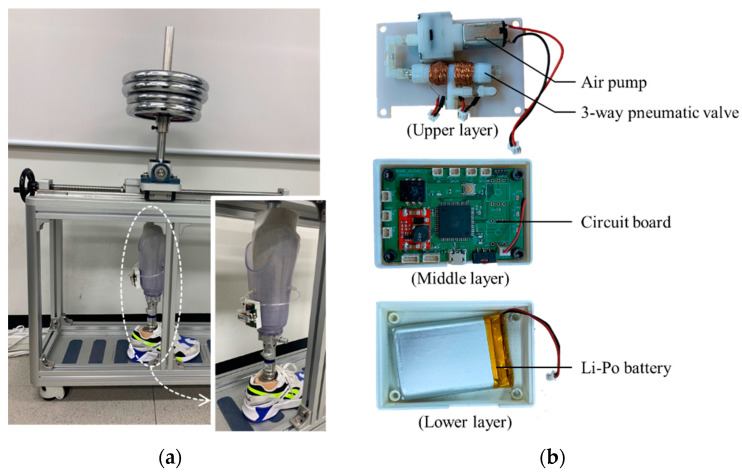
(**a**) The view of the prosthetic socket in the gait simulator; (**b**) the inside view of the portable control device.

The control device can be attached to the prosthetic socket as shown in Figure 17a. Figure 17b shows the inside view of the control device which an air pump and a 3-way pneumatic valve (upper layer), a Li-Po battery (lower layer) and a circuit board (middle layer). As an air pump, we used a commercial air mini pump of YYPN20 (Huizhou Yingyi Motor Co. Ltd., Guangdong, China) with a maximum pressure of 60 kPa.

**Figure 18:**
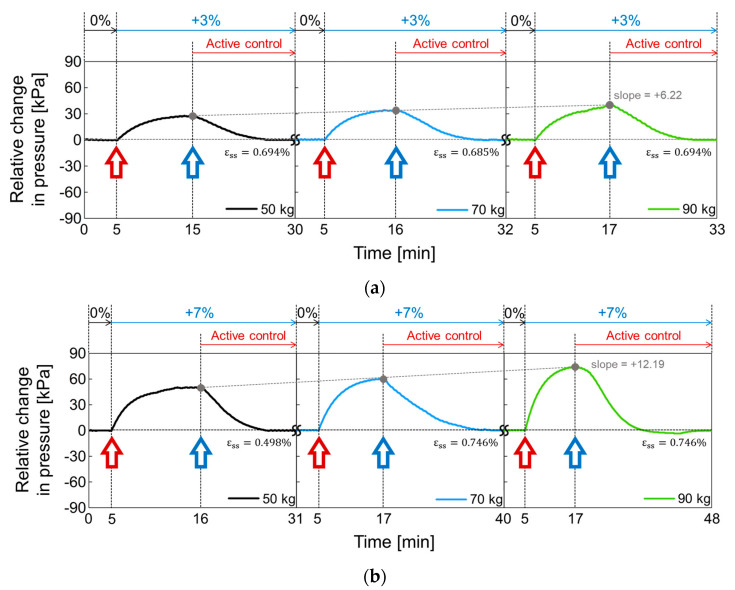

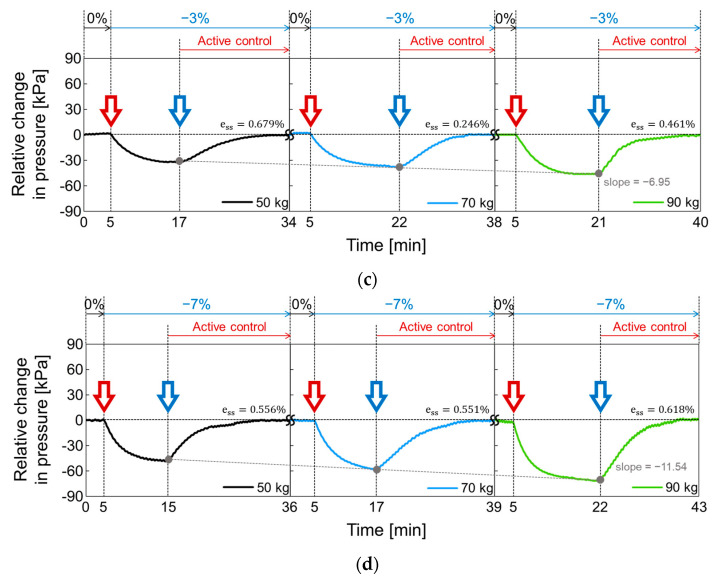
The active volume compensation of the proposed socket when the weight is 50, 70 and 90 kg and the limb volume changes (**a**) from 0% to +3%; (**b**) from 0% to +7%; (**c**) from 0% to -3%; (**d**) from 0% to -7%.

In Figure 18, the active volume compensation of the proposed socket was verified during the repetitive gait cycle. The relative changes in pressure inside the socket were evaluated using the weight loads of 50, 70, and 90 kg and the residual limb models with volume change rates of +3%, −3%, 0%, +7%, and −7%. The relative change in pressure represents the difference between the initial pressure and the changed pressure. The limb model with a volume change of 0% is the reference for the limb model. In Figure 18, the red open arrow indicates when the reference limb is changed to a different limb model, and the blue open arrow indicates the start of the active control completing a closed loop. For the first 5 min, the gait was performed with the reference limb. The residual limb was then replaced with a different limb model, and the pressure began to increase or decrease after the replacement. After that, when the pressure value was stabilized, the active control was performed, and the pressure value was returned to the initial state thanks to the active control. These procedures were repeated while changing the weight load. Figure 18a shows the relative change in socket pressure when the residual limb was changed from 0% to +3% limb model. Without active control, the value of the maximum pressure change increases with a slope of 6.22 as the weight gradually increases. In Figure 18b, the limb model of +7% was used, and maximum values in the pressure change have a slope of 12.19 with different weights. This slope becomes larger when the volume change of the residual limb is larger. Additionally, the relative pressure change decreases when using the −3% limb model as shown in Figure 18c and further decreases under heavy weight conditions. In Figure 18d, when the lower limb model is −7%, the minimum values of the pressure change has a slope of −11.54 as the weight increases, whereas the slope is −6.95 at −3% limb model of Figure 18c.

To evaluate the performance of active control in the socket system, we analyzed the steady state error ($\varepsilon_{ss}$), not a transient response, because the socket system operates very slowly. When the volume change of the residual limb was from −7% to + 7%, the steady state error was less than 0.75%. The proposed socket system successfully compensated for volume changes in the residual limb. Thus, even if the volume of the residual limb was changed, the pressure in the prosthetic socket remained the same.

## 4. Conclusions

In this study, an active prosthetic socket was developed to compensate for the volume change of the residual limb. The proposed socket uses an inflatable air bladder to monitor the internal pressure of the socket and keep the pressure distribution uniform and constant while walking. If the internal pressure of the socket changes due to a change in limb volume, the initial pressure state is restored through inflation or deflation of the air bladders. The injection or exhaust of air is through a 3-way pneumatic valve. Since the 3-way pneumatic valve is a latching type, the plunger keeps its position by the magnetic force without the electric power supply. In the paper, the electromagnetic properties were analyzed to find the optimum number of coil turns for the solenoid to move the permanent magnet plunger. A hysteresis control algorithm was proposed to keep the pressure in the socket close to the initial set point over a long period of time. In experiments, the active volume compensation of the socket was successfully verified during repetitive gait cycle using the weight loads of 50, 70, and 90 kg and the residual limb models with volume change rates of +3%, -3%, 0%, +7%, and -7%. As a result, it was confirmed that the pressure of the residual limb recovered to the initial state after the active control. It achieves a steady state error of less than 0.75% even if the volume of the residual limb is changed from - 7% to + 7 %. In the future, through clinical trials, this socket system could potentially be applied as patient-specific prosthesis and contribute to improving the patient’s quality of life.

## Data Availability

The data presented in this study are available on request from the corresponding author.
